# Development and Characterization of Novel Polymorphic Microsatellite Markers for *Tapinoma indicum* (Hymenoptera: Formicidae)

**DOI:** 10.1093/jisesa/ieab047

**Published:** 2021-07-23

**Authors:** Li Yang Lim, Abdul Hafiz Ab Majid

**Affiliations:** Household & Structural Urban Entomology Laboratory, Vector Control Research Unit, School of Biological Sciences, Universiti Sains Malaysia, Penang, 11800 Minden, Malaysia

**Keywords:** ant, nuisance pest, molecular biology, microsatellite marker, polymorphism

## Abstract

*Tapinoma indicum* (Forel) (Hymenoptera: Formicidae) is a nuisance pest in Asia countries. However, studies on *T. indicum* are limited, especially in the field of molecular biology, to investigate the species characteristic at the molecular level. This paper aims to provide valuable genetic markers as tools with which to study the *T. indicum* population. In this study, a total of 143,998 microsatellite markers were developed based on the 2.61 × 10^6^ microsatellites isolated from *T. indicum* genomic DNA sequences. Fifty selected microsatellite markers were amplified with varying numbers of alleles ranging from 0 to 19. Seven out of fifty microsatellite markers were characterized for polymorphism with the Hardy–Weinberg equilibrium (HWE) and linkage disequilibrium (LD) analysis. All seven microsatellite markers demonstrated a high polymorphic information content (PIC) value ranging from 0.87 to 0.93, with a mean value of 0.90. There is no evidence of scoring errors caused by stutter peaks, no large allele dropout, and no linkage disequilibrium among the seven loci; although loci Ti-Tr04, Ti-Tr09, Ti-Te04, Ti-Te13, and Ti-Pe5 showed signs of null alleles and deviation from the HWE due to excessive homozygosity. In conclusion, a significant amount of microsatellite markers was developed from the data set of next-generation sequencing, and seven of microsatellite markers were validated as informative genetic markers that can be utilized to study the *T. indicum* population.

More than 2,693 species and 7,953 morphospecies of ants have been identified worldwide from published and unpublished data (Gibb et al. 2017). Although only around 20 species of ants are considered pests, the National Pest Management Association ranks ants as the number one nuisance pest in America (NPMA 2012). According to the 2020 State of the Ant Control Market survey, ant control services contributed an average of 22.2% of company revenue in 2019, more than any other pest control service (Syngenta 2020). A recent study found 13 species of ants, belonging to eight genera and three subfamilies, in residential areas of Penang Island, Malaysia, and *Tapinoma indicum* F. was found to be one of the most abundant (Ab Majid et al. 2016). *T. indicum*, described by Forel in 1895 and also known as the ghost ant, is a widely distributed pest in Asian countries. This species is a source of irritation and disruption to humans. It often constructs nests and forages in buildings for food sources.

Previous studies on *T. indicum* were mainly focused on feeding preferences and foraging activities (Chong and Lee 2006), plant-derived pesticides (Lim and Ab Majid 2019), and bait preferences (Lee 2008, Chong and Lee 2009, Ab Majid et al. 2018). To date, no molecular research has been conducted on *T. indicum*. Only one set of *T. indicum* mitochondrial cytochrome c oxidase subunit 1 (CO1) sequences is published in the National Center for Biotechnology Information (NCBI); however, these sequences were not discussed or included in the published paper (Wang et al. 2018).

Microsatellite markers are an essential tool in molecular biology. The highly polymorphic characteristic of microsatellite markers makes them a useful genetic marker. They are widely used in various applications, including genetic diversity studies, phylogeographic studies, and population genetic structure and phylogenetic analysis to provide detailed information that stands as a baseline to study on the social structure of the pest (Goropashnaya et al. 2007, Hick and Marshall 2018, Trible et al. 2020). These species-specific markers also can be used to determine the origin and dispersal routes of insect pest which contribute to a more efficient insect pest management. Unfortunately, no microsatellite marker has been reported for *T. indicum* despite its prevalence as a nuisance pest. Thus, this paper aims to identify *T. indicum* species-specific microsatellite markers using the data obtained from the Illumina next-generation sequencing platform. This paper also aims to validate seven selected microsatellite markers polymorphism with Hardy–Weinberg equilibrium (HWE) and linkage disequilibrium (LD) analysis. By providing this new set of useable genetic markers, the population genetic structure, genetic diversity, and breeding patterns can now be analyzed to increase our understanding of *T. indicum* and aid in tracking and designing control strategies for this important pest.

## Materials and Methods

### Microsatellite Marker Design

As previously described, the whole-genome sequencing dataset of *T. indicum* deposited in the National Centre for Biotechnology Information (NCBI) under Bioproject PRJNA598521 was used to identify microsatellite markers (Lim and Ab Majid 2020). The microsatellite marker design method was improved by Seri Masran and Ab Majid (2018). The microsatellite from the dataset was screened using Msatcommander v1.0.8 (Faircloth 2008) with minimum repeats for each type of motifs; eight mononucleotide repeats, eight dinucleotide repeats, eight trinucleotide repeats, six tetranucleotide repeats, six pentanucleotide repeats, and six hexanucleotide repeats. The primer pairs flanking the isolated microsatellites were designed using Primer3Plus (Untergasser et al. 2012). The primers were designed within the size range of 18–22 bp; the melting temperature and GC content were set within the ranges of 58–62°C and 30–70%, respectively.

### Sample Collection and DNA Extraction

The sample collection data of *T. indicum* are listed in Table 1. The genomic DNA of *T. indicum* was extracted based on the manufacturer’s protocol with minimal modifications using the HiYield Plus Genomic DNA Mini Kit (Blood/Tissue/Cultured Cells) (Real Biotech Corp., Taipei, Taiwan). The genomic DNA from a worker ant from each collection site was exclusively isolated from the head to minimize the potential DNA extraction interference caused by the microbes living in the thorax and abdomen. Thus, the DNA extraction elution step was modified to maximize the yield of the extracted DNA. The head tissue was vortexed in lysis buffer with Proteinase K and incubated at 60°C for 1 h. The elution process was carried out using 50 μl elution buffer after the binding of the DNA and filter column through ethanol washing. The elution step was repeated using the eluate from the first elution step. In total, 50 μl DNA samples were collected and validated using NanoDrop 2000c (Thermo Fisher Scientific, MA).

**Table 1.** Details of *T. indicum* sample collection

Abbreviation	Collection site	No. of samples	Collection date	Latitude	Longitude
R01	Balik Pulau, Penang	8	24 June 2019	N 5⁰22′22.60318ʺ	E 100⁰13′5.87651ʺ
R02	Balik Pulau, Penang	8	25 June 2019	N 5⁰21′7.2ʺ	E 100⁰14′18.84ʺ
R03	Balik Pulau, Penang	8	25 June 2019	N 5⁰21′2.0953ʺ	E 100⁰13′51.56244ʺ
R04	Balik Pulau, Penang	8	27 June 2019	N 5⁰20′42.22365ʺ	E 100⁰13′47.66868ʺ
R05	Balik Pulau, Penang	8	27 June 2019	N 5⁰20′33.76815ʺ	E 100⁰13′41.02418ʺ
S01	Gelugor, Penang	8	16 Mar. 2019	N 5⁰21′0.64874ʺ	E 100⁰18′26.67501ʺ
S02	Gelugor, Penang	8	18 Mar. 2019	N 5⁰20′59.20185ʺ	E 100⁰18′2.37219ʺ
S03	Gelugor, Penang	8	18 Mar. 2019	N 5⁰20′58.182ʺ	E 100⁰17′50.052ʺ
S04	Gelugor, Penang	8	21 Mar. 2019	N 5⁰21′7.84953ʺ	E 100⁰18′26.11859ʺ
S05	Gelugor, Penang	8	24 Mar. 2019	N 5⁰20′41.96047ʺ	E 100⁰17′41.08443ʺ
U01	George Town, Penang	8	19 Aug. 2019	N 5⁰25′4.524ʺ	E 100⁰19′34.662ʺ
U02	George Town, Penang	8	19 Aug. 2019	N 5⁰25′8.118ʺ	E 100⁰19′37.494ʺ
U03	George Town, Penang	8	20 Aug. 2019	N 5⁰25′43.452ʺ	E 100⁰19′5.874ʺ
U04	George Town, Penang	8	20 Aug. 2019	N 5⁰25′42.612ʺ	E 100⁰19′2.556ʺ
U05	George Town, Penang	8	04 Sept. 2019	N 5⁰24′36.31004ʺ	E 100⁰19′11.42311ʺ

### Microsatellite Marker Validation

Fifty microsatellite markers were selected for the polymorphism analysis based on the length and type of repeat motif and the penalty score. In total, 14 microsatellite markers for each dinucleotide, trinucleotide, and tetranucleotide motif, and six microsatellite markers for pentanucleotide motif were selected, as shown in Table 2. The microsatellite markers were used to amplify 45 pooling *T. indicum* worker genomes, which comprised three individuals from each location (Table 1). The PCR reaction mixture contained 12.5 µl master mix of brand DNA polymerase, 5 µl of DNA, 5.5 µl of distilled water, and 1 µl of each primer (0.4 µM). The PCR thermocycler profile was set at 94°C for 10 min during the initial denaturation stage, followed by 35 cycles for 30 s at 94°C for the denaturation phase, 30 s at 60°C for the annealing phase, and 1 min at 72°C for the extension phase. The PCR ended with the final extension phase at 72°C for 10 min, before being held at 4°C. The PCR product was visualized using agarose gel electrophoresis. Fragment analysis was carried out using the Fragment Analyzer Automated CE System (Agilent Technologies, CA) (Seri Masran and Ab Majid 2018). The number of alleles was scored with the Prosize 3.0 software package (Agilent Technologies, CA).

**Table:** Table 2. Information of the 50 selected microsatellite markers developed from *T. indicum*

Locus	Repeat Motif	Primer (5’-3’)	Number of alleles
Ti-Di01	(AG)_44_	F = CGTGCGTATGTAGAAGCGTG	10
		R = TCCCTCTCTTTCTACGACCC	
Ti-Di02	(AG)_28_	F = GTCGCGGATGGAATGTCTTG	19
		R = ACTTATCCGGGTGCACTCTC	
Ti-Di03	(AG)_28_	F = AGCAGCGGAGAAATCAAAGC	6
		R = GTCCCTCGCGCTGATTAAAC	
Ti-Di04	(AG)_33_	F = ATGGTGGAACGACGGATAGG	8
		R = CGCCACAGAGAGAGATACCC	
Ti-Di05	(AG)_29_	F = CACTGTGAGAGGGTAGGACG	10
		R = AGTGCCTCAGGAGTCAGTTG	
Ti-Di06	(AG)_30_	F = AAGTCCACCATCGACCGTAG	12
		R = AGCGAACATTGTTTCCCGTC	
Ti-Di07	(AG)_27_	F = TCACCTTTGATGCGCGATTC	7
		R = CATGTATTTCCGCCCACACC	
Ti-Di08	(AG)_47_	F = AATCGATGTTAGCTCGTGGG	12
		R = CTCCCTCACATCCACACTCG	
Ti-Di09	(AG)_46_	F = ATTGATGGCGGTAGAGAGGG	2
		R = TCGTTCTCCTTCCTCCACG	
Ti-Di10	(AG)_42_	F = AGAGCGAAATCAAAGGCAGC	6
		R = TGATACGCATGTTGTTGGCC	
Ti-Di11	(AC)_47_	F = GGTGAAGGTGAAGGGAAAGG	0
		R = TCACTCTCTTTCCTCCTTGTTG	
Ti-Di12	(AT)_26_	F = TCCTGGTGCTTTATCTCGGG	1
		R = TCTACGCAACAGGTCAGTCC	
Ti-Di13	(AC)_45_	F = CGTGATTTCGCATACCCTTCC	2
		R = TGTGTACGTTCTACTTATCGGG	
Ti-Di14	(AC)_38_	F = CAAAGGTTGTGTGAGCCAAC	0
		R = CAACACTGTCACGCTCGC	
Ti-Tr01	(ACG)_24_	F = GAGGAGAGGTGAAGTCGTCG	8
		R = CCATTTCCCTCTGCGTATCG	
Ti-Tr02	(ACG)_24_	F = ATCCCTCCCATTTCCCTCTG	6
		R = GAGGAGAGGTGAAGTCGTCG	
Ti-Tr03	(ACG)_24_	F = AACTTTCTCGGTTCAACGCC	10
		R = CGGACGAAGGTTGTTAGTAGTG	
Ti-Tr04	(AAG)_19_	F = AGGGCTTCTTCTTCCACGAG	10
		R = GGCTCTCTCGGCTCGTTG	
Ti-Tr05	(CCG)_20_	F = GCATACACGTAGCGGCAG	0
		R = GGACTATCTTTACGGTGCCC	
Ti-Tr06	(AAG)_19_	F = CGTCCTCGTATCCTGTTTCAAG	0
		R = CCGCCGGTAGAGGTTCTAG	
Ti-Tr07	(ACG)_27_	F = GAAAGAGTGCAGGACCATGC	0
		R = CTTCCTCGTCTCGTCCCG	
Ti-Tr08	(ACG)_23_	F = AGTCGCGTTGGTCATTGTTC	0
		R = ATCATCGTCTACAGCGGCG	
Ti-Tr09	(CCG)_18_	F = CACGGGCTTAGGAATTATAGCG	9
		R = TAATGCGCGTCGTAGGTAGG	
Ti-Tr10	(CCG)_20_	F = CGAGGCATACACGTAGCGG	11
		R = GTACATTAGATGCGGTGAAACG	
Ti-Tr11	(ACG)_20_	F = TTATCCTCGAGCGTTCCACC	9
		R = TCCGTTGAAATTTGGCCGAC	
Ti-Tr12	(ACG)_22_	F = GGCGCGTGATAGTCGTACC	11
		R = CCGTGTGCTCTTCCGATCTG	
Ti-Tr13	(ACG)_22_	F = ACTCTCTTCCTTTCCGCTCAG	0
		R = GGACCGTACAGAAGAGGACG	
Ti-Tr14	(ACG)_21_	F = ATATTGTGTTCCCGTGGCAG	2
		R = GGAGCTGCATATTCCGTTGG	
Ti-Te01	(AGCC)_18_	F = TAGCTGCTTGGTTTGCTTGC	2
		R = CTGGGTTAACTTTCCGACCG	
Ti-Te02	(ACGC)_20_	F = TAACCTTTCAAACGACCGGC	1
		R = GATCGAGTGTGTGTACCGAC	
Ti-Te03	(AAGG)_17_	F = GCAGGAAGGGAGGGAAGG	12
		R = TGGTCTCTTCACACGGACG	
Ti-Te04	(ACGC)_14_	F = GTGTGCGGGAGTGTTCTTC	6
		R = TTCGGCTTGATGTGTTGTGC	
Ti-Te05	(AGCC)_14_	F = CTGGGTTAACTTTCCGACCG	12
		R = TAGCTCCTTGGTTTGCTTGC	
Ti-Te06	(ACGC)_16_	F = GATTCGCGTGGGTGGTTTC	11
		R = GGCGAAACTTTCCTCTCGTC	
Ti-Te07	(AGGC)_14_	F = CATCCTGCGCGATACAACG	0
		R = CTTGTCTGCTTGCCTGCC	
Ti-Te08	(AGCC)_17_	F = CTGGGTTAACTTTCCGACCG	0
		R = TAGCTGCTTGGTTTGCTTGC	
Ti-Te09	(ACGC)_16_	F = CTTTCCTCTCGTCTCGCCG	0
		R = GATTCGCGTGGGTGGTTTC	
Ti-Te10	(ACGC)_14_	F = TTCGGCTTGATGTGTTGTGC	0
		R = TGTGTGCGGGAGTGTTCTTC	
Ti-Te11	(AGCC)_14_	F = GGGTTAACTTTCCGACCGG	10
		R = TAGCTGCTTGGTTTGCTTGC	
Ti-Te12	(AGGC)_14_	F = CTTGTCTGCTTGCCTGCC	10
		R = TTCGCACGAATCCCAGACAG	
Ti-Te13	(ACGC)_13_	F = ACAAGTAGACGGCTCCCTTC	14
		R = CGGGCTAATCGATGGTTTCTC	
Ti-Te14	(ACGC)_14_	F = TGTTCTTCACTGCAGGCG	8
		R = TTCGGCTTGATGTGTTGTGC	
Ti-Pe01	(AACCT)_12_	F = CTCCGAAACAGCTCTACACG	1
		R = GCGAGTTTGAATCTGGTAAGGG	
Ti-Pe02	(AGGCG)_11_	F = CACCTATAGATCTGCTGCGTG	2
		R = CTTGTCTCGTCTCGCCTCG	
Ti-Pe03	(ACGAG)_10_	F = GTCTCGTCTCGTTCCGTCC	2
		R = AGGCGAAACGAGACGAGAC	
Ti-Pe04	(AGGCG)_12_	F = CTTGTCTCGTCTCGCCTCG	12
		R = GGGCGCTCACCAACCTATAG	
Ti-Pe05	(ACGCC)_8_	F = GGAGAGATCCTGGAACCGTG	4
		R = ATTTGGGAGTCTGGGTTGGC	
Ti-Pe06	(AACCT)_9_	F = CGCGAGTTTGAATTTGGTAAGG	10
		R = CACAGCTACCACCCACATCC	
Ti-Pe07	(AACCT)_9_	F = CTCCGAAACAGCTCTACACG	0
		R = CGCGAGTTTGAATTTGGTAAGG	
Ti-Pe08	(AGGCG)_9_	F = TAGGGTATGGCATGGCAAGG	0
		R = CTCACCCTTCAACCGCCC	

In total, seven microsatellite markers for different motif types were selected for the polymorphism test. The PCR reactions were performed on the individual genomic DNA from each location with five replicates (Table 1). Micro-Checker v2.2.0.3 was used to check the fragment analysis results, as related to the observed and expected null allele frequencies, to detect any errors that might have occurred in PCR, such as failure amplification, stuttering, or large allele dropout (Van Oosterhout et al. 2004). Allele frequency analysis was then conducted, including observed and expected heterozygosity, the number of alleles, and the polymorphic information content (PIC), using Cervus v3.0.7 (Kalinowski et al. 2007). HWE and LD were analyzed using GENEPOP v4.7 with a dememorization number of 1,000, 100 batches, and 1,000 iterations per batch (Raymond and Rousset 1995, Rousset 2008).

## Results and Discussion

In total, 2.61 × 10^6^ microsatellites were screened, including 1.43 × 10^6^ mononucleotide microsatellites, 1.08 × 10^6^ dinucleotide microsatellites, 5.40 × 10^4^ trinucleotide microsatellites, 2.19 × 10^4^ tetranucleotide microsatellites, 2.35 × 10^4^ pentanucleotide microsatellites, and 527 hexanucleotide microsatellites following the previous 3.27 × 10^7^ raw sequence reads (Lim and Ab Majid 2020). In total, 143,998 primer pairs flanking the screened microsatellites, including 82,145 mononucleotide microsatellite markers, 56,131 dinucleotide microsatellite markers, 4,186 trinucleotide microsatellite markers, 1,357 tetranucleotide microsatellite markers, 163 pentanucleotide microsatellite markers, and 16 hexanucleotide microsatellite markers, were designed. Supplement 1 provides a complete list of the 143,998 primer pairs with details. The use of next-generation sequencing technology enables the large-scale collection of genomic data. This technology enables researchers to identify a significant number of microsatellite markers at a relatively low per-locus cost. Unlike other design techniques, a limited number of microsatellite markers were developed for Formicidae (Kronauer and Gadau 2002, Pearcy et al. 2004, Arthofer et al. 2007, Lemos et al. 2020).

From the pooled genome of *T. indicum,* fifty selected microsatellite markers were successfully amplified with various allele counts, with PCR settings ranging from 0 to 19 alleles, as shown in Table 2. Mononucleotide microsatellite markers were excluded from primer selection due to the high error rate during amplification of the mononucleotide repeat sequences by PCR (Shinde 2003, Baptiste et al. 2015). The low quantity of hexanucleotide microsatellite markers was also excluded. A gradient PCR was used to optimize the PCR protocol and to determine the optimal annealing temperature for the microsatellite markers. All of the identified microsatellite markers are expected to perform well using the same PCR protocol as the designed markers, i.e., at a similar size range, melting temperature, and GC content.

Out of the 50 microsatellite markers, seven microsatellite markers were selected for further validation based on the type of motif and amplification effectiveness. The selected microsatellite markers are dinucleotide motif: Ti-Di02 and Ti-Di06; trinucleotide motif: Ti-Tr04 and Ti-Tr09; tetranucleotide motif: Ti-Te04 and Ti-Te13; and pentanucleotide motif: Ti-Pe05. The seven selected microsatellite markers successfully genotyped a total of 75 individuals from 15 different locations. These seven microsatellite markers proved to be functional, i.e., they were not hard to score, nor demonstrated monomorphic or amplification failures.

Seven selected microsatellite markers resulted in high genetic diversity, producing 14–24 alleles per locus from 15 populations with five replicates, as shown in Table 3. They provided sufficient information for genetic studies of *T. indicum*. The PIC value ranging from 0.87 to 0.93 (mean = 0.9015) indicated that the microsatellite markers of *T. indicum* in this study possessed the desired polymorphism characteristics, as a PIC value of more than 0.5 indicates high diversity (Table 3).

**Table 3.** Number of alleles amplified by seven microsatellite markers in each population and the result of the polymorphism analysis with HWE and the LD test

Locus	Number of alleles																PIC	H0	He	F(Null)	HWE (Y/N)	LD (Y/N)
	R01	R02	R03	R04	R05	S01	S02	S03	S04	S05	U01	U02	U03	U04	U05	Total						
Ti-Di02	7	7	7	6	6	7	8	6	8	6	10	6	8	4	7	23	0.9224	1.0000	0.9332	−0.0388	Y	N
Ti-Di06	7	7	7	5	7	4	3	6	4	5	7	6	6	4	5	16	0.8945	0.8533	0.9088	0.0267	Y	N
Ti-Tr04	4	1	3	4	4	3	3	3	5	3	5	4	3	3	3	16	0.8866	0.0000	0.9017	1.0000	N	N
Ti-Tr09	4	4	3	4	3	3	4	3	5	4	3	5	5	1	4	16	0.8901	0.0933	0.9047	0.8112	N	N
Ti-Te04	4	5	3	4	2	3	6	2	3	4	3	4	3	3	4	24	0.9169	0.0667	0.9280	0.8659	N	N
Ti-Te13	4	6	6	4	6	7	2	2	5	6	6	7	4	5	4	23	0.9302	0.5600	0.9403	0.2494	N	N
Ti-Pe05	3	4	5	4	3	4	5	3	4	4	4	5	5	3	4	14	0.8700	0.0000	0.8859	1.0000	N	N
Mean	4.71	4.86	4.86	4.43	4.43	4.43	4.43	3.57	4.86	4.57	5.43	5.29	4.86	3.29	4.43	18.86	0.9015	0.3676	0.9147	0.5592	N	N

Table 3 summarizes the details of the conducted analysis. There is no evidence of scoring errors due to stuttering peaks or large allele dropout in the fragment analysis. However, Ti-Tr04, Ti-Tr09, Ti-Te04, Ti-Te13, and Ti-Pe5 represent the null alleles and deviated from the HWE. Ti-Di02 and Ti-Di06 did not demonstrate null alleles, and they were in statistical accordance with the HWE. A high level of homozygosity was observed in Ti-Tr04, Ti-Tr09, Ti-Te04, Ti-Te13, and Ti-Pe5. The homozygous excess caused the markers to show signs of null alleles and depart from the HWE. This study revealed a similar result to that of Zima et al. 2016, who detected a high level of homozygosity for the *Tapinoma melanocephalum* microsatellite markers. The excess of homozygosity may arise from biological processes, such as selection, the founder effect, bottleneck, or inbreeding (Goodisman and Hahn 2005). There was no linkage disequilibrium found among any of the loci (Table 3).

This study presents 143,998 new microsatellite markers, provides a wide range of markers for future research. Further studies concerning cross-species amplification can also be conducted with the large pool of developed microsatellite markers (Butler et al. 2014). Seven microsatellite markers, including Ti-Di02, Ti-Di06, Ti-Tr04, Ti-Tr09, Ti-Te04, Ti-Te13, and Ti-Pe5, were validated as effective markers with high polymorphic characteristic ranging from 0.87 to 0.93 (mean = 0.9015) and are ready to be used to analyze the *T. indicum* population genetic structure.
