# Corrigendum to: Development and Characterization of Novel Polymorphic Microsatellite Markers for *Tapinoma indicum* (Hymenoptera: Formicidae)

**DOI:** 10.1093/jisesa/ieab083

**Published:** 2021-11-02

**Authors:** Li Yang Lim, Abdul Hafiz Ab Majid

**Affiliations:** Household & Structural Urban Entomology Laboratory, Vector Control Research Unit, School of Biological Sciences, Universiti Sains Malaysia, Penang, 11800 Minden, Malaysia

Correction of “Li Yang Lim and Abdul Hafiz Ab Majid. 2021. Development and Characterization of Novel Polymorphic Microsatellite Markers for *Tapinoma indicum* (Hymenoptera: Formicidae). J. Insect Sci.”

DOI: 10.1093/jisesa/ieab047

There was an error in the primer code in the last two paragraphs of the “Results and Discussions” section when this paper was first published. The correct primer code should read as **“Ti-Di06”** instead of **“Ti-Di05”**.

When this paper was first published Table 2 was incorrect. The table originally appeared as:

**Table 2:** Information of the 50 selected microsatellite markers developed from *T. indicum*

Locus	Repeat motif	Primer (5′–3′)	Number of alleles
Ti-Di01	(AG)_44_	F = CGTGCGTATGTAGAAGCGTG	10
		R = TCCCTCTCTTTCTACGACCC	
Ti-Di02	(AG)_33_	F = ATGGTGGAACGACGGATAGG	8
		R = CGCCACAGAGAGAGATACCC	
Ti-Di03	(AG)_28_	F = AGCAGCGGAGAAATCAAAGC	6
		R = GTCCCTCGCGCTGATTAAAC	
Ti-Di04	(AG)_28_	F = GTCGCGGATGGAATGTCTTG	19
		R = ACTTATCCGGGTGCACTCTC	
Ti-Di05	(AG)_29_	F = CACTGTGAGAGGGTAGGACG	10
		R = AGTGCCTCAGGAGTCAGTTG	
Ti-Di06	(AG)_30_	F = AAGTCCACCATCGACCGTAG	12
		R = AGCGAACATTGTTTCCCGTC	
Ti-Di07	(AG)_27_	F = TCACCTTTGATGCGCGATTC	7
		R = CATGTATTTCCGCCCACACC	
Ti-Di08	(AG)_47_	F = AATCGATGTTAGCTCGTGGG	12
		R = CTCCCTCACATCCACACTCG	
Ti-Di09	(AG)_46_	F = ATTGATGGCGGTAGAGAGGG	2
		R = TCGTTCTCCTTCCTCCACG	
Ti-Di10	(AG)_42_	F = AGAGCGAAATCAAAGGCAGC	6
		R = TGATACGCATGTTGTTGGCC	
Ti-Di11	(AC)_47_	F = GGTGAAGGTGAAGGGAAAGG	0
		R = TCACTCTCTTTCCTCCTTGTTG	
Ti-Di12	(AT)_26_	F = TCCTGGTGCTTTATCTCGGG	1
		R = TCTACGCAACAGGTCAGTCC	
Ti-Di13	(AC)_45_	F = CGTGATTTCGCATACCCTTCC	2
		R = TGTGTACGTTCTACTTATCGGG	
Ti-Di14	(AC)_38_	F = CAAAGGTTGTGTGAGCCAAC	0
		R = CAACACTGTCACGCTCGC	
Ti-Tr01	(ACG)_24_	F = GAGGAGAGGTGAAGTCGTCG	8
		R = CCATTTCCCTCTGCGTATCG	
Ti-Tr02	(ACG)_24_	F = ATCCCTCCCATTTCCCTCTG	6
		R = GAGGAGAGGTGAAGTCGTCG	
Ti-Tr03	(ACG)_24_	F = AACTTTCTCGGTTCAACGCC	10
		R = CGGACGAAGGTTGTTAGTAGTG	
Ti-Tr04	(ACG)_20_	F = TTATCCTCGAGCGTTCCACC	9
		R = TCCGTTGAAATTTGGCCGAC	
Ti-Tr05	(CCG)_20_	F = GCATACACGTAGCGGCAG	0
		R = GGACTATCTTTACGGTGCCC	
Ti-Tr06	(AAG)_19_	F = CGTCCTCGTATCCTGTTTCAAG	0
		R = CCGCCGGTAGAGGTTCTAG	
Ti-Tr07	(ACG)_27_	F = GAAAGAGTGCAGGACCATGC	0
		R = CTTCCTCGTCTCGTCCCG	
Ti-Tr08	(ACG)_23_	F = AGTCGCGTTGGTCATTGTTC	0
		R = ATCATCGTCTACAGCGGCG	
Ti-Tr09	(ACG)_22_	F = GGCGCGTGATAGTCGTACC	11
		R = CCGTGTGCTCTTCCGATCTG	
Ti-Tr10	(CCG)_20_	F = CGAGGCATACACGTAGCGG	11
		R = GTACATTAGATGCGGTGAAACG	
Ti-Tr11	(AAG)_19_	F = AGGGCTTCTTCTTCCACGAG	10
		R = GGCTCTCTCGGCTCGTTG	
Ti-Tr12	(CCG)_18_	F = CACGGGCTTAGGAATTATAGCG	9
		R = TAATGCGCGTCGTAGGTAGG	
Ti-Tr13	(ACG)_22_	F = ACTCTCTTCCTTTCCGCTCAG	0
		R = GGACCGTACAGAAGAGGACG	
Ti-Tr14	(ACG)_21_	F = ATATTGTGTTCCCGTGGCAG	2
		R = GGAGCTGCATATTCCGTTGG	
Ti-Te01	(AGCC)_18_	F = TAGCTGCTTGGTTTGCTTGC	2
		R = CTGGGTTAACTTTCCGACCG	
Ti-Te02	(ACGC)_20_	F = TAACCTTTCAAACGACCGGC	1
		R = GATCGAGTGTGTGTACCGAC	
Ti-Te03	(AAGG)_17_	F = GCAGGAAGGGAGGGAAGG	12
		R = TGGTCTCTTCACACGGACG	
Ti-Te04	(ACGC)_16_	F = GATTCGCGTGGGTGGTTTC	11
		R = GGCGAAACTTTCCTCTCGTC	
Ti-Te05	(AGCC)_14_	F = CTGGGTTAACTTTCCGACCG	12
		R = TAGCTCCTTGGTTTGCTTGC	
Ti-Te06	(ACGC)_14_	F = GTGTGCGGGAGTGTTCTTC	6
		R = TTCGGCTTGATGTGTTGTGC	
Ti-Te07	(AGGC)_14_	F = CATCCTGCGCGATACAACG	0
		R = CTTGTCTGCTTGCCTGCC	
Ti-Te08	(AGCC)_17_	F = CTGGGTTAACTTTCCGACCG	0
		R = TAGCTGCTTGGTTTGCTTGC	
Ti-Te09	(ACGC)_16_	F = CTTTCCTCTCGTCTCGCCG	0
		R = GATTCGCGTGGGTGGTTTC	
Ti-Te10	(ACGC)_14_	F = TTCGGCTTGATGTGTTGTGC	0
		R = TGTGTGCGGGAGTGTTCTTC	
Ti-Te11	(AGCC)_14_	F = GGGTTAACTTTCCGACCGG	10
		R = TAGCTGCTTGGTTTGCTTGC	
Ti-Te12	(AGGC)_14_	F = CTTGTCTGCTTGCCTGCC	10
		R = TTCGCACGAATCCCAGACAG	
Ti-Te13	(ACGC)_13_	F = ACAAGTAGACGGCTCCCTTC	14
		R = CGGGCTAATCGATGGTTTCTC	
Ti-Te14	(ACGC)_14_	F = TGTTCTTCACTGCAGGCG	8
		R = TTCGGCTTGATGTGTTGTGC	
Ti-Pe01	(AACCT)_12_	F = CTCCGAAACAGCTCTACACG	1
		R = GCGAGTTTGAATCTGGTAAGGG	
Ti-Pe02	(AGGCG)_11_	F = CACCTATAGATCTGCTGCGTG	2
		R = CTTGTCTCGTCTCGCCTCG	
Ti-Pe03	(ACGAG)_10_	F = GTCTCGTCTCGTTCCGTCC	2
		R = AGGCGAAACGAGACGAGAC	
Ti-Pe04	(AGGCG)_12_	F = CTTGTCTCGTCTCGCCTCG	12
		R = GGGCGCTCACCAACCTATAG	
Ti-Pe05	(AACCT)_9_	F = CGCGAGTTTGAATTTGGTAAGG	10
		R = CACAGCTACCACCCACATCC	
Ti-Pe06	(ACGCC)_8_	F = GGAGAGATCCTGGAACCGTG	4
		R = ATTTGGGAGTCTGGGTTGGC	
Ti-Pe07	(AACCT)_9_	F = CTCCGAAACAGCTCTACACG	0
		R = CGCGAGTTTGAATTTGGTAAGG	
Ti-Pe08	(AGGCG)_9_	F = TAGGGTATGGCATGGCAAGG	0
		R = CTCACCCTTCAACCGCCC	

The full and correct Table 2 is reproduced below.

**Table 2:** Information of the 50 selected microsatellite markers developed from *T. indicum*

Locus	Repeat Motif	Primer (5’-3’)	Number of alleles
Ti-Di01	(AG)_44_	F = CGTGCGTATGTAGAAGCGTG	10
		R = TCCCTCTCTTTCTACGACCC	
Ti-Di02	(AG)_28_	F = GTCGCGGATGGAATGTCTTG	19
		R = ACTTATCCGGGTGCACTCTC	
Ti-Di03	(AG)_28_	F = AGCAGCGGAGAAATCAAAGC	6
		R = GTCCCTCGCGCTGATTAAAC	
Ti-Di04	(AG)_33_	F = ATGGTGGAACGACGGATAGG	8
		R = CGCCACAGAGAGAGATACCC	
Ti-Di05	(AG)_29_	F = CACTGTGAGAGGGTAGGACG	10
		R = AGTGCCTCAGGAGTCAGTTG	
Ti-Di06	(AG)_30_	F = AAGTCCACCATCGACCGTAG	12
		R = AGCGAACATTGTTTCCCGTC	
Ti-Di07	(AG)_27_	F = TCACCTTTGATGCGCGATTC	7
		R = CATGTATTTCCGCCCACACC	
Ti-Di08	(AG)_47_	F = AATCGATGTTAGCTCGTGGG	12
		R = CTCCCTCACATCCACACTCG	
Ti-Di09	(AG)_46_	F = ATTGATGGCGGTAGAGAGGG	2
		R = TCGTTCTCCTTCCTCCACG	
Ti-Di10	(AG)_42_	F = AGAGCGAAATCAAAGGCAGC	6
		R = TGATACGCATGTTGTTGGCC	
Ti-Di11	(AC)_47_	F = GGTGAAGGTGAAGGGAAAGG	0
		R = TCACTCTCTTTCCTCCTTGTTG	
Ti-Di12	(AT)_26_	F = TCCTGGTGCTTTATCTCGGG	1
		R = TCTACGCAACAGGTCAGTCC	
Ti-Di13	(AC)_45_	F = CGTGATTTCGCATACCCTTCC	2
		R = TGTGTACGTTCTACTTATCGGG	
Ti-Di14	(AC)_38_	F = CAAAGGTTGTGTGAGCCAAC	0
		R = CAACACTGTCACGCTCGC	
Ti-Tr01	(ACG)_24_	F = GAGGAGAGGTGAAGTCGTCG	8
		R = CCATTTCCCTCTGCGTATCG	
Ti-Tr02	(ACG)_24_	F = ATCCCTCCCATTTCCCTCTG	6
		R = GAGGAGAGGTGAAGTCGTCG	
Ti-Tr03	(ACG)_24_	F = AACTTTCTCGGTTCAACGCC	10
		R = CGGACGAAGGTTGTTAGTAGTG	
Ti-Tr04	(AAG)_19_	F = AGGGCTTCTTCTTCCACGAG	10
		R = GGCTCTCTCGGCTCGTTG	
Ti-Tr05	(CCG)_20_	F = GCATACACGTAGCGGCAG	0
		R = GGACTATCTTTACGGTGCCC	
Ti-Tr06	(AAG)_19_	F = CGTCCTCGTATCCTGTTTCAAG	0
		R = CCGCCGGTAGAGGTTCTAG	
Ti-Tr07	(ACG)_27_	F = GAAAGAGTGCAGGACCATGC	0
		R = CTTCCTCGTCTCGTCCCG	
Ti-Tr08	(ACG)_23_	F = AGTCGCGTTGGTCATTGTTC	0
		R = ATCATCGTCTACAGCGGCG	
Ti-Tr09	(CCG)_18_	F = CACGGGCTTAGGAATTATAGCG	9
		R = TAATGCGCGTCGTAGGTAGG	
Ti-Tr10	(CCG)_20_	F = CGAGGCATACACGTAGCGG	11
		R = GTACATTAGATGCGGTGAAACG	
Ti-Tr11	(ACG)_20_	F = TTATCCTCGAGCGTTCCACC	9
		R = TCCGTTGAAATTTGGCCGAC	
Ti-Tr12	(ACG)_22_	F = GGCGCGTGATAGTCGTACC	11
		R = CCGTGTGCTCTTCCGATCTG	
Ti-Tr13	(ACG)_22_	F = ACTCTCTTCCTTTCCGCTCAG	0
		R = GGACCGTACAGAAGAGGACG	
Ti-Tr14	(ACG)_21_	F = ATATTGTGTTCCCGTGGCAG	2
		R = GGAGCTGCATATTCCGTTGG	
Ti-Te01	(AGCC)_18_	F = TAGCTGCTTGGTTTGCTTGC	2
		R = CTGGGTTAACTTTCCGACCG	
Ti-Te02	(ACGC)_20_	F = TAACCTTTCAAACGACCGGC	1
		R = GATCGAGTGTGTGTACCGAC	
Ti-Te03	(AAGG)_17_	F = GCAGGAAGGGAGGGAAGG	12
		R = TGGTCTCTTCACACGGACG	
Ti-Te04	(ACGC)_14_	F = GTGTGCGGGAGTGTTCTTC	6
		R = TTCGGCTTGATGTGTTGTGC	
Ti-Te05	(AGCC)_14_	F = CTGGGTTAACTTTCCGACCG	12
		R = TAGCTCCTTGGTTTGCTTGC	
Ti-Te06	(ACGC)_16_	F = GATTCGCGTGGGTGGTTTC	11
		R = GGCGAAACTTTCCTCTCGTC	
Ti-Te07	(AGGC)_14_	F = CATCCTGCGCGATACAACG	0
		R = CTTGTCTGCTTGCCTGCC	
Ti-Te08	(AGCC)_17_	F = CTGGGTTAACTTTCCGACCG	0
		R = TAGCTGCTTGGTTTGCTTGC	
Ti-Te09	(ACGC)_16_	F = CTTTCCTCTCGTCTCGCCG	0
		R = GATTCGCGTGGGTGGTTTC	
Ti-Te10	(ACGC)_14_	F = TTCGGCTTGATGTGTTGTGC	0
		R = TGTGTGCGGGAGTGTTCTTC	
Ti-Te11	(AGCC)_14_	F = GGGTTAACTTTCCGACCGG	10
		R = TAGCTGCTTGGTTTGCTTGC	
Ti-Te12	(AGGC)_14_	F = CTTGTCTGCTTGCCTGCC	10
		R = TTCGCACGAATCCCAGACAG	
Ti-Te13	(ACGC)_13_	F = ACAAGTAGACGGCTCCCTTC	14
		R = CGGGCTAATCGATGGTTTCTC	
Ti-Te14	(ACGC)_14_	F = TGTTCTTCACTGCAGGCG	8
		R = TTCGGCTTGATGTGTTGTGC	
Ti-Pe01	(AACCT)_12_	F = CTCCGAAACAGCTCTACACG	1
		R = GCGAGTTTGAATCTGGTAAGGG	
Ti-Pe02	(AGGCG)_11_	F = CACCTATAGATCTGCTGCGTG	2
		R = CTTGTCTCGTCTCGCCTCG	
Ti-Pe03	(ACGAG)_10_	F = GTCTCGTCTCGTTCCGTCC	2
		R = AGGCGAAACGAGACGAGAC	
Ti-Pe04	(AGGCG)_12_	F = CTTGTCTCGTCTCGCCTCG	12
		R = GGGCGCTCACCAACCTATAG	
Ti-Pe05	(ACGCC)_8_	F = GGAGAGATCCTGGAACCGTG	4
		R = ATTTGGGAGTCTGGGTTGGC	
Ti-Pe06	(AACCT)_9_	F = CGCGAGTTTGAATTTGGTAAGG	10
		R = CACAGCTACCACCCACATCC	
Ti-Pe07	(AACCT)_9_	F = CTCCGAAACAGCTCTACACG	0
		R = CGCGAGTTTGAATTTGGTAAGG	
Ti-Pe08	(AGGCG)_9_	F = TAGGGTATGGCATGGCAAGG	0
		R = CTCACCCTTCAACCGCCC	

These errors have now been corrected online.

